# Spectrum of congenital heart disease in children admitted for cardiac surgery at Rehman Medical Institute, Peshawar, Pakistan

**DOI:** 10.12669/pjms.291.2910

**Published:** 2013

**Authors:** Shahzada Bakhtyar Zahid, Anwer Zeb Jan, Samreen Ahmed, Humaira Achakzai

**Affiliations:** 1 Dr. Shahzada Bakhtyar Zahid, MRCPCH, Department of Pediatrics, Rehman Medical Institute, Peshawar, Pakistan.; 2 Dr. Anwer Zeb Jan, MRCP, Department of Pediatrics, Rehman Medical Institute, Peshawar, Pakistan.; 3 Dr. Samreen Ahmed, FCPS, Department of Pediatrics, Rehman Medical Institute, Peshawar, Pakistan.; 4 Dr. Humaira Achakzai, FCPS, Department of Medicine, Rehman Medical Institute, Peshawar, Pakistan.

**Keywords:** Congenital heart disease, Cyanotic and acyanotic heart disease, Ventricular septal defect, Patent Ductus Arteriosus, Tetralogy of Fallot

## Abstract

***Objective:*** To assess age, gender distribution and relative frequency of congenital heart disease (CHD) in children who underwent palliative or corrective cardiac surgery at Rehman Medical Institute, Peshawar.

***Methodology:*** This retrospective study was conducted in Department of Cardiac Surgery at Rehman Medical Institute (RMI), Peshawar from May 2008 till May 2010. One hundred and twenty three patients up to age group of 16 years with confirmed diagnosis of congenital heart disease admitted for Cardiac Surgery at Rehman Medical Institute, Peshawar were included.

***Results:*** Out of 123 patients, there were 71 males (57.7%) and 52 females (42.2%), with male to female ratio of 1.3:1. Sixty five (52.8%) of the total cases had acyanotic heart defects. Cyanotic heart defects were seen in 58 patients (47.1%). Ventricular Septal Defect (VSD) followed by Patent Ductus Arteriosus (PDA) and Atrial Septal Defect (ASD), were the commonest acyanotic heart lesions, 33.8%, 23.0% and 16.9% respectively. Tetralogy of Fallot (TOF) was the commonest cyanotic lesion.

***Conclusion*** *:* Majority of patients with congenital heart disease had acyanotic CHD with the commonest lesion being VSD. Tetralogy Of Fallot (TOF) was the commonest cyanotic lesion. Most of the patients were less than five years with no significant difference in sex distribution. Availability of expertise locally will lead to more patients getting surgical treatment at an earlier age thereby reducing morbidity and mortality and improving quality of life for these children.

## Introduction

 Congenital heart disease by definition is the structural or functional heart disease, present at birth, even if it is detected later on.^1^ In Pakistan majority of the childbirths still takes place at home and routine neonatal screening is not common, so it is very difficult to calculate true birth prevalence of congenital heart disease.^2^

 Congenital heart disease is the most common congenital problem effecting nearly 25% of all children with congenital malformation^3^ with an incidence of almost 8/1000 live births.^4^ Early diagnosis and prompt treatment has great implication on prognosis and can result in significant decrease in morbidity and mortality. The relatively high birth rate in India, an average of 150,000 children are born with congenital abnormalities with 50,000 requiring surgery in the first year of life and only one thousand cardiac surgeries are done in the early infancy.^5^ The relevant high mortality rate due to CHD is due to the fact that many of the babies who could be saved by surgery do not have the chance due to high cost and inadequate facilities; the situation is the same in other South Asian countries including Pakistan.

 Despite vast improvements in medical field, congenital heart disease is still one of the leading cause of death and can present in different age groups from birth to adolescence.^6^ The presentation of children with CHD can be very variable; they may be asymptomatic and discovered only accidentally when a murmur is noted during check up for an unrelated illness or routine neonatal checkup,^7^ or may present with symptoms like cyanosis, clubbing of fingers nails to full blown congestive heart failure.^6^^,^^7^ CHD has not been studied thoroughly in Pakistan as in other western and neighboring countries.

 The purpose of this study was to present a single center experience in CHD at RMI, Peshawar, which is the biggest cardiac tertiary care center in the province, receiving patients not only from all over Pakistan but also Afghanistan and Central Asia and to compare it with studies done in other countries and similar studies locally.

## Methodology

 This is a retrospective chart review conducted at cardiac surgical unit from May 2008 to May 2010 at Rehman Medical Institute, Peshawar, Pakistan. All children with confirmed diagnosis of CHD admitted for corrective or palliative cardiac surgery were included. A total of 123 children were studied with age ranging from one month to 16 years.

**Table-I:** Age Distribution of 123 Cases

*Age group*	*No of cases*	*Percentage (%)*
1-5 years	47	38.2
6-10 years	41	32.5
11-16 years	37	29.6

 Clinical data was reviewed. Consideration was given to the total number of cases with CHD, age, sex distribution and type of CHD. Patients with multiple heart anomalies or syndromes were also included. Patients with acquired heart diseases, such as rheumatic heart disease, were not included.

## Results

 A total of 123 children were included. The data from these patients were evaluated regarding sex distribution, age and relative frequency of different congenital heart defects. There were 71 males (57.7%) and 52 females (42.2%), with male to female ratio of 1.3**:**1. The age ranged from one month to 16 years. Afghani patients constituted 60% of the population studied while 40% cases reported from Khyber Pukhtoonkhwa province.

**Table-II:** Relative distribution of Cyanotic and non cyanotic heart lesions

*Cardiac lesion*	*Number*	*Percentage *
Acyanotic		
Ventricular septal defect alone	22	33.8
Patent ductus arteriosus	15	23
Atrial septal defect	11	16.9
VSD with PDA	10	15.3
VSD with ASD	3	4.6
Aortic valve stenosis	2	3
Coarctation of aorta	2	3
Cyanotic		
Tetralogy of Fallot	30	51.7
Complex CHD	6	10.3
Transposition of great arteries	10	31.3
VSD with RVOT	5	8.6
Complete AV septal defect	6	10.3
Pulm stenosis + Total anomalous venous return	1	1.7
Fallots pentology	1	1.7

**Table-III:** Comparative study of the lesions with other studies

*Type of CHD*	*RMI, Peshawar*	*Prince Hashem Hospital*	*Fuad Abbag (Saudi Arabia)*	*Aiberta Hertiage Pediatric cardiology program (Canada)*	*Mary M K Shann (Taiwan)*
VSD	33.8%	43.4%	32.5%	34.6%	39.3%
ASD	16.9%	13.3%	10.4%	10.5%	5.3%
PDA	23%	8.3	15.8%	10.8%	9.8%
PS	---	6.2%	10.1%	--	2.5%
AS	3%	4.3%	2.7%	--	-
Coarctation of aorta	3%	3.4%	3.3%	--	1.1%
Tetralogy of Fallot	51.7%	9.5%	4.5%	10.2%	12.3%
Complex CHD	10.3%	2.25%	2.7%	3.5%	5.0%
Transposition of great arteries	31.3%	5.5%	1.5%	5.1%	5.3%
Complete AV septal defect	10.3%	3.6%	--	4.49%	--

 Sixty five (52.8%) of the total cases had acyanotic heart defects. Cyanotic heart defects were seen in 58 patients (47.1%).Ventricular septal defect (VSD) followed by Patent ductus arteriosus and atrial septal defect (ASD), were the commonest acyanotic heart lesions, 33.8%, 23.0% and 16.9% respectively. While combination of VSD with other anomalies like PDA and ASD were 13(20%), there were two cases of Aortic stenosis and Coarctation of aorta. In Cyanotic heart defects TOF was the commonest 30 (51.7%), followed by Transposition of Great Arteries (TGA) 10(31.3%), complex heart disease 6(10.3%), five cases each of Atrioventricular canal defect (AV canal defect) and VSD with RVOT obstruction. There was one case of pulmonary stenosis with total anomalous venous return and fallot’s pentology each. There was male predominance in both cyanotic and acyanotic heart lesions. However, complex heart defects and mixed cardiac lesions like VSD with Right ventricular outflow tract obstruction (RVOT obstruction) and VSD with ASD were more common in females.

## Discussion

 CHD is an important group of diseases that cause great morbidity & mortality in children.^8^ Our study is confined to Rehman Medical Institute only where not only Pakistani but Afghani patients are also treated so it does not give true incidence and prevalence of CHD in total population.

 It is accepted that the advancement in health sciences has helped in early diagnosis, attention and awareness among general pediatrician and early referral to pediatric cardiologists has resulted in an increase of reported prevalence of CHD.^6^^,^^8^ The present study indicates that CHD is an important pediatric cardiac problem. There were 123 cases of CHD 71 were males (57.7%) and 52(42.2%) females. Male to female ratio is 1.3:1. The male predominance is similar to other studies done in Pakistan. In the study at Pediatric Dept of JPMC, Karachi by Rahimtoola males was 57% and females 43%.^9^ Acyanotic heart defects were present in 52.8% of cases while 47.1% had cyanotic heart disease, correlating with a study carried out in Jordan Department of Pediatrics at Prince Hashim Military Hospital Jordan-Zarka.^10^

 Maximum number of children with CHD was observed in 1-5 years and 6-10 years age groups. Same was observed in the study done by AL-e-Hag, 1994.^11^ There was male predominance in both cyanotic and acyanotic heart lesions. However, complex heart defects and mixed cardiac lesions like VSD with RVOT obstruction and VSD with ASD were more common in females. This finding is the same as already reported in a previously done study^12^ but it is contradictory to the finding made by Burki MK and Babar GS^13^ who reported both sexes to be equally affected. This difference may be due to a number of factors such as socio-economic, cultural, ecological and genetic factors.

VSD is found to be the most common acyanotic CHD (33.8%) in our study. This is in accordance to what is reported in other studies as shown in Table-III.^14^^,^^15^ Worldwide, VSD is the most common acyanotic CHD accounting for 25-30% of all CHD.^16^ PDA ranked second in frequency accounting for 23% of the acyanotic heart defects which is higher than found in other studies. Similarly cyanotic heart defects were in higher percentage as compared to others studies shown in Table-III, with TOF and TGA dominating the list with (51.7%) and (31.1%) respectively, probably because most of the cases had reported for surgical repair to the Pediatric cardiac surgical unit. While number of cases of VSD, ASD, AS and Coarctation of aorta is comparable to international studies done in Canada, Saudi Arabia and Taiwan^14^^,^^15^ and also to a local study by Rehman F et al.^2^

## Conclusions

 The study gives an overview of the pattern of congenital heart disease in children admitted for cardiac surgery. In order to avoid complications, reduce mortality and improve quality of life, earlier detection and correction of disease is of utmost importance. Many lesions are amenable to surgery, availability of local expertise and awareness amongst parents and professionals will help do these at the optimal time.
